# Resolution of chronic idiopathic urticaria with setmelanotide in a patient with Bardet-Biedl Syndrome: A case report

**DOI:** 10.1016/j.obpill.2025.100221

**Published:** 2025-11-03

**Authors:** Kate Haggerty, Jennifer Black, Ruth Abeles

**Affiliations:** a Perlman Clinic Wellness, Volunteer Faculty, University of California, San Diego, USA; b Obesity and Internal Medicine Physician, Perlman Clinic Wellness, USA; c Center for Advanced Weight Management, UCSD Bariatric and Metabolic Institute, USA

**Keywords:** Bardet-biedl syndrome, Setmelanotide, Chronic idiopathic urticaria, MC4R, Case report

## Abstract

**Introduction:**

This case describes the resolution of refractory chronic idiopathic urticaria (CIU) with setmelanotide in a patient with Bardet-Biedl Syndrome (BBS). While setmelanotide is approved for syndromic obesity, this report highlights a novel anti-inflammatory effect potentially mediated by melanocortin-4 receptor (MC4R) agonism, expanding current understanding of its immunomodulatory role in mast cell–mediated disease.

**Main symptoms and clinical findings:**

The patient presented with lifelong hyperphagia and class III obesity beginning in childhood, along with multiple features fulfilling BBS clinical criteria: visual impairment with night vision difficulties, renal abnormalities with proteinuria, irregular menses with polycystic ovaries, and neurodevelopmental delay. She also reported refractory CIU with pruritic hives occurring several times weekly despite dual antihistamine therapy. On examination, BMI was 40.5 kg/m^2^, and dermatologic evaluation confirmed recurrent hives unresponsive to standard treatment.

**Diagnosis:**

Genetic testing revealed a heterozygous pathogenic *BBS9* variant (c.1120C > T; p. Arg374∗), supporting a BBS diagnosis. Secondary causes of urticaria were excluded. Setmelanotide was initiated at 2 mg daily and titrated to 3 mg, leading to complete urticaria resolution within three weeks and cessation of antihistamines. Symptoms recurred during a brief treatment pause and resolved upon reinitiation, demonstrating a temporal association. Hyperphagia scores improved from 20 to 0, with significant quality-of-life gains. Adjunct phentermine 18.75 mg daily was later added to optimize appetite control. After ten months, urticaria remained in sustained remission, with only one isolated cold-induced episode.

**Conclusion:**

Setmelanotide produced durable remission of refractory CIU in a patient with BBS, suggesting MC4R agonism may exert immunomodulatory effects beyond weight regulation. This finding underscores the broader potential of precision therapies for rare obesity syndromes to reveal new mechanisms of inflammation control.

## Graphical abstract

**Figure:**
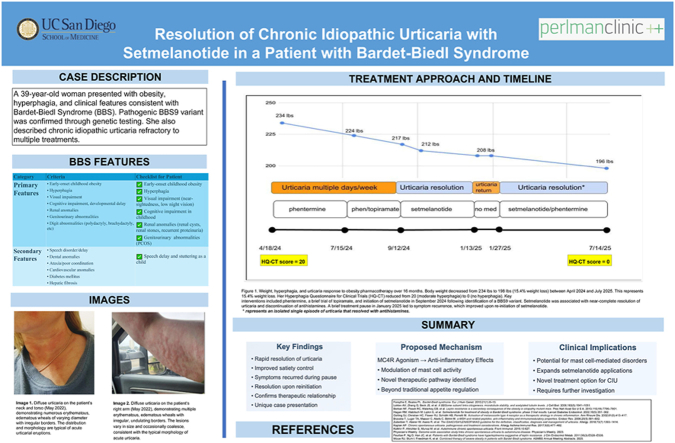

## 1 Introduction

Bardet-Biedl Syndrome (BBS) is an autosomal recessive rare ciliopathy characterized by early-onset obesity, hyperphagia, retinal dystrophy, polydactyly, renal anomalies, and neurodevelopmental impairment, with an estimated prevalence of 1 in 100,000 to 160,000 individuals [1,2]. The clinical diagnosis of BBS is made by the presence of either four major features or three major features and two minor features established by Beales et al. [1,2]. Pathogenic variants in more than 26 genes, including *BBS9*, disrupt the BBSome complex responsible for trafficking G protein–coupled receptors (GPCRs) to primary cilia, leading to hypothalamic dysfunction and impaired satiety regulation [[3], [4], [5]]. Setmelanotide, a melanocortin-4 receptor (MC4R) agonist, is FDA-approved for obesity related to BBS and has demonstrated significant improvements in appetite control and weight outcomes in clinical trials [[6], [7], [8]]. While its role in syndromic obesity is established, emerging evidence suggests that MC4R agonism may extend beyond energy homeostasis to modulation of immune responses and inflammation [[9], [10], [11]].

Resolution of refractory chronic idiopathic urticaria (CIU) was observed in a patient with genetically-supported Bardet-Biedl syndrome (BBS) following treatment with setmelanotide. CIU is a mast cell–mediated disorder that often persists despite high-dose antihistamines and may require biologics or immunosuppressants in refractory cases [14,15]. The patient's urticaria remitted rapidly after initiating setmelanotide, recurred during a treatment interruption, and again resolved upon reinitiation, strongly suggesting a causal link. To our knowledge, this is the first reported case of CIU remission associated with MC4R agonist therapy. These findings highlight a novel potential anti-inflammatory role for setmelanotide, supporting the hypothesis that melanocortin signaling may regulate mast cell activity and broaden its therapeutic utility in immune-mediated disease [12,13].

## 2 Patient information

### 2.1 De-identified patient-specific information

The patient is a 39-year-old woman with childhood-onset obesity and hyperphagia who presented as a new patient for evaluation of syndromic obesity and longstanding refractory chronic idiopathic urticaria (CIU). Family history was notable for obesity and metabolic disorders, though no known ciliopathies were reported.

### 2.2 Primary concerns and symptoms of the patient

Her symptoms included poor satiety control, progressive weight gain, and recurrent diffuse, pruritic hives occurring several times per week despite continuous dual antihistamine therapy. She reported a history of learning delays, speech disorder in childhood, visual impairment with night vision difficulties , renal abnormalities, and irregular menses. Psychosocially, she described frustration and reduced quality of life due to the daily burden of urticaria, as well as longstanding challenges with weight management despite consistent dietary efforts.

### 2.3 Medical, family, and psychosocial history, including relevant genetic information

The patient is a 39-year-old woman with childhood-onset obesity and lifelong hyperphagia, consistent with syndromic obesity, who also has a history of learning delays, childhood speech disorder, visual impairment with night vision difficulties, renal abnormalities with renal cysts and proteinuria, irregular menses, and polycystic ovaries with mildly elevated androgens. She has experienced longstanding refractory chronic idiopathic urticaria since 2021, occurring multiple times per week despite dual antihistamine therapy, which significantly reduced her quality of life and contributed to frustration, depression, and self-doubt after years of unexplained medical issues often attributed solely to lifestyle factors. Family history was notable for obesity and metabolic disorders, but no known ciliopathies were reported. Genetic testing through the *Uncovering Rare Obesity* program identified a heterozygous pathogenic variant in BBS9 (c.1120C > T; p. Arg374∗), supporting the diagnosis of Bardet-Biedl Syndrome, along with two variants of uncertain significance in KIDINS220 and *POMC*, the clinical contributions of which remain unclear. The genetic counselor provided through Prevention Genetics noted that pathogenic variants in BBS9 cause autosomal recessive Bardet-Biedl syndrome 9. Although only one pathogenic allele was identified, a second undetected variant may be present.

### 2.4 Relevant past interventions with outcomes

Prior to initiating setmelanotide, the patient underwent multiple unsuccessful interventions for both obesity and chronic idiopathic urticaria. She trialed several anti-obesity medications, including phentermine, orlistat, metformin, bupropion, and a short course of topiramate, none of which produced durable weight loss or meaningful appetite control. Her insurance coverage denied GLP1 receptor agonist medications, and it was cost-prohibitive for her to pay out of pocket for these. For urticaria, she was maintained on daily antihistamine therapy, which provided only partial symptom relief and required continuous use due to frequent, diffuse, pruritic hives. Despite these measures, she remained significantly symptomatic with persistent hyperphagia and recurrent urticaria until genetic testing identified a pathogenic variant in BBS9, supporting a diagnosis o**f** Bardet-Biedl Syndrome and ultimately guiding initiation of setmelanotide therapy.

## 3 Clinical findings: significant physical exam and important clinical findings

On examination, the patient's weight was 236 lbs with a BMI of 40.5 kg/m^2^, consistent with longstanding class III obesity. Phenotypic features included night vision difficulty, learning delays, childhood speech disorder, renal abnormalities, and irregular menses. Dermatologic evaluation revealed recurrent diffuse pruritic hives occurring multiple times per week, despite daily diphenhydramine and cetirizine therapy. These clinical findings aligned with syndromic obesity and chronic idiopathic urticaria refractory to standard antihistamine treatment.

## 4 Timeline: Historical and current information from this episode of care organized as a timeline

In April 2024, she was evaluated in the Obesity Medicine clinic and started on phentermine, followed by a brief topiramate trial in July 2024, which was discontinued due to side effects. Genetic testing later identified a pathogenic BBS9 variant, confirming Bardet-Biedl Syndrome. In September 2024, she initiated setmelanotide at 2 mg daily, titrated to 3 mg, leading to complete urticaria resolution within three weeks and discontinuation of antihistamines. She remained hive-free until January 2025, when treatment was paused due to an injection site reaction, during which both hyperphagia and urticaria returned. After resuming setmelanotide, symptoms again resolved, and she has since remained in remission. In February 2025, phentermine 18.75 mg daily was reintroduced as adjunct therapy, further improving appetite control. By April 2025, only a single cold-induced urticaria episode had occurred, and as of June 2025, she had sustained remission with no further recurrences while on therapy.

**Fig. 1:**
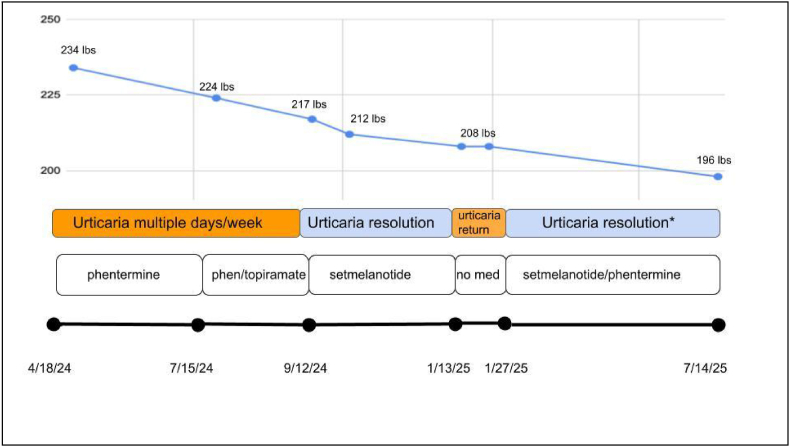
Weight and urticaria response to obesity pharmacotherapy over 12 months. Body weight decreased from 234 lbs to 196 lbs between April 2024 and July 2025. Key interventions included phentermine, a brief trial of topiramate, and initiation of setmelanotide in September 2024 following identification of a BBS9 variant. Setmelanotide was associated with near-complete resolution of urticaria and discontinuation of antihistamines. A brief treatment pause in January 2025 led to symptom recurrence, which improved upon re-initiation of setmelanotide and phentermine. Urticaria frequency declined from several times weekly to approximately once monthly. ∗ *represents an isolated single episode of urticaria that resolved with antihistamines*.

See Fig. 1 for Timeline Summary and Supplement Materials for patient rash.

## 5 Diagnostic assessment

### 5.1 Diagnostic testing

Diagnostic evaluation included laboratory studies that demonstrated mildly elevated androgens and an increased urine protein/creatinine ratio, while other metabolic and autoimmune panels were unremarkable. Pelvic ultrasound showed polycystic ovaries with mildly elevated androgens. Her renal ultrasound showed a non-descript renal “mass” that was further evaluated with a CT with and without contrast and found to be a renal cyst and renal stones. She had been evaluated by urology for recurrent proteinuria and an elevated urine protein/creatinine ratio and found to have a normal cystoscopy. An ophthalmology evaluation has been ordered to further assess reported night vision difficulties, but remains pending due to insurance-related delays.

At the initial visit, all patients evaluated in the obesity medicine clinic undergo a standardized screening process that includes three targeted questions: a history of childhood-onset obesity, whether they have ever had a body mass index (BMI) greater than 40 kg/m^2^, and whether they have perceived their hunger to be excessive compared with peers or family members. Patients who screen positive are subsequently assessed using Bardet-Biedl Syndrome (BBS) clinical diagnostic criteria and are offered participation in the *Uncovering Rare Obesity* genetic testing program.

Genetic testing was performed using the patient's saliva sample, which was submitted through the *Uncovering Rare Obesity* program and analyzed via next-generation sequencing by Prevention Genetics, LLC. Three variants were identified. The first was a heterozygous pathogenic variant in *BBS9* (c.1120C > T; p. Arg374∗), which results in premature protein termination and has been previously reported in individuals with Bardet-Biedl Syndrome [16]. The second was a heterozygous variant of uncertain significance (VUS) in KIDINS220 (c.4970G > A; p. Ser1657Asn). Although truncating and missense variants in KIDINS220 have been observed in individuals with obesity, the gene-disease relationship remains inconclusive. The third variant was a heterozygous VUS in *POMC* (c.394C > G; p. Pro132Ala). This variant has been identified in several individuals with obesity [[17], [18], [19], [20], [21]], but has also been observed in control populations [21], limiting its interpretive significance. A summary of the genetic results is presented in Table 2. Genetic consultation confirmed that although only one pathogenic allele was identified, a second undetected variant may be present. Further genetic counseling was offered to this patient as well to discuss her results.

Hyperphagia was assessed using the Hyperphagia Questionnaire for Clinical Trials (HQ-CT). At present, there is no consensus regarding the most appropriate hyperphagia assessment tool for MC4R pathway–associated disorders [22]. We found the questionnaire employed by Haws et al. to be aligned with the characterization and grading of hyperphagia in this context [23], consistent with ongoing efforts to refine hyperphagia definitions and measurement strategies for improved diagnosis and management of MC4R pathway–related disease [22]. The patient's HQ-CT score was 20, consistent with moderate hyperphagia.

### 5.2 Diagnostic challenges (access, financial or cultural)

In this case, diagnostic challenges were driven by access barriers, insurance/referral delays, and gaps in clinical awareness. The patient had seen multiple providers over many years, yet her obesity and hyperphagia were often attributed solely to lifestyle factors, and testing for rare genetic causes of obesity is not typically well known or pursued in the primary care setting. Although genetic testing through the *Uncovering Rare Obesity* program assisted with the diagnosis of Bardet-Biedl Syndrome, such testing is not universally accessible and may present financial challenges for patients outside of sponsored programs. Insurance-related delays also hindered timely follow-up diagnostics, particularly ophthalmologic and genetic evaluation.

Clinical manifestations of Bardet-Biedl syndrome (BBS) are highly variable, an issue that is being further elucidated as pathogenic variants are identified in a larger proportion of affected individuals. This variability can be diagnostically challenging, as it has even been reported that some individuals who are homozygous or compound heterozygous for BBS-associated variants do not meet established clinical diagnostic criteria [2]. Additionally, genomic testing can identify disease-causing variants in known genes that are not yet part of standard gene panels, as well as in previously unrecognized genes that may be linked to BBS [2].

In considering the differential diagnosis, several syndromic obesity disorders were evaluated. Alström syndrome shares features of obesity, retinal dystrophy, and metabolic abnormalities but typically presents with sensorineural hearing loss and cardiomyopathy, which were not observed in this patient. Cohen syndrome, another rare disorder associated with developmental delay, retinal dystrophy, and neutropenia, was also considered but was less consistent given the absence of characteristic craniofacial features and hematologic findings. The patient's irregular menses, polycystic ovaries, and elevated androgens are consistent with polycystic ovary syndrome (PCOS), which may coexist as a comorbidity rather than serve as the primary explanation for her obesity and hyperphagia. Chronic urticaria was extensively evaluated to exclude secondary causes; autoimmune, allergic, and systemic triggers were not identified, supporting the diagnosis of chronic idiopathic urticaria. Proteinuria raised concern for early renal involvement, which is a recognized feature of Bardet-Biedl Syndrome. While she was noted to have renal cysts and urolithiasis on imaging, her renal function appeared preserved on laboratory evaluation and her proteinuria had resolved upon retesting. Finally, although only one pathogenic allele was identified, a second undetected variant may be present. Also, while variants of uncertain significance in KIDINS220 and *POMC* were identified, their clinical contribution remains uncertain; ongoing monitoring is warranted given reported associations of *POMC* variants with obesity and of KIDINS220 variants with neurodevelopmental features. Ultimately, the presence of a pathogenic *BBS9* variant in conjunction with the patient's clinical phenotype supports Bardet-Biedl Syndrome as the unifying diagnosis.

### 5.3 Diagnosis and other diagnoses considered

The patient's presentation of early-onset obesity, hyperphagia, visual impairment, renal abnormalities, irregular menses, and neurodevelopmental delays, together with genetic support of a pathogenic BBS9 variant (c.1120C > T; p. Arg374∗), established the diagnosis of Bardet-Biedl Syndrome (BBS) as the unifying condition. Her recurrent hives were classified as chronic idiopathic urticaria (CIU) after extensive evaluation excluded autoimmune, allergic, and systemic causes. The differential diagnosis included Alström syndrome, which shares obesity and retinal dystrophy but typically presents with sensorineural hearing loss and cardiomyopathy not observed in this patient, and Cohen syndrome, which involves developmental delay and retinal dystrophy but was less consistent given the absence of characteristic craniofacial or hematologic features. Polycystic ovary syndrome (PCOS), suggested by irregular menses, polycystic ovaries, and elevated androgens, was considered a comorbidity rather than the primary explanation for obesity and hyperphagia. Proteinuria raised concern for early renal involvement, a known feature of BBS, although preserved renal function argued against primary glomerulopathy. Finally, variants of uncertain significance in KIDINS220 and *POMC* were noted, though their clinical contribution remains uncertain. Ultimately, the pathogenic BBS9 variant and clinical phenotype supported Bardet-Biedl Syndrome as the unifying diagnosis.

## 6 Therapeutic intervention

### 6.1 Types of therapeutic intervention (pharmacologic)

At the time of her initial evaluation in the Obesity Medicine clinic in April 2024, the patient reported daily use of diphenhydramine 100 mg and cetirizine 10 mg to manage chronic intermittent urticaria, which occurred multiple times per week. Obesity pharmacotherapy was initiated with phentermine on April 18, 2024, followed by a brief trial of topiramate in July 2024, which was discontinued due to intolerable side effects. Upon identification of a heterozygous pathogenic variant in the BBS9 gene, the patient was approved for setmelanotide therapy, which was initiated in September 2024 at 2 mg subcutaneously daily and titrated to 3 mg daily after two weeks. Within three weeks of starting setmelanotide, urticaria resolved completely, and the patient discontinued all antihistamines. She remained symptom-free until January 2025, when she paused therapy due to a painful local injection site reaction. During the treatment break, both hyperphagia and urticaria returned with notable intensity. After restarting setmelanotide, her symptoms again resolved fully. Aside from a single episode of cold-induced urticaria on April 16, 2025 (week 32), she had no further episodes while on active therapy. Phentermine was reintroduced in February 2025 at a dose of 18.75 mg daily. On dual therapy, the patient's Hyperphagia Questionnaire for Clinical Trials (HQ-CT) score decreased from 20 to 0, and she continued to report complete remission of hyperphagic symptoms. As of her most recent follow-up in June 2025, urticaria episodes had decreased from multiple occurrences per week to only one isolated episode over a 10-month treatment period, demonstrating durable control on setmelanotide. This is depicted in Fig. 1.

### 6.2 Administration of therapeutic intervention (dose, strength and duration)

The patient was approved for setmelanotide therapy following identification of a pathogenic BBS9 variant. Treatment was initiated in September 2024 at a dose of 2 mg subcutaneously once daily, and after two weeks, the dose was titrated to 3 mg daily. Within three weeks of initiation, her chronic idiopathic urticaria resolved completely, allowing discontinuation of antihistamines. She continued therapy with sustained remission over a 10-month period, with a brief treatment pause in January 2025 due to a painful local injection site reaction, during which urticaria recurred. Upon restarting setmelanotide at the same daily subcutaneous dose, her symptoms resolved fully and remained controlled. Additionally, phentermine 18.75 mg daily was reintroduced in February 2025 as adjunct therapy.

### 6.3 Changes in therapeutic intervention (with rationale)

In January 2025, therapy was temporarily paused due to a painful local injection site reaction, during which hyperphagia and urticaria recurred. Upon reinitiation at the same daily dose, symptoms resolved fully, and the reaction did not recur. In February 2025, adjunct therapy with phentermine 18.75 mg daily was added, resulting in further improvement in appetite regulation, with the Hyperphagia Questionnaire score decreasing from 20 to 0. The patient has since continued on dual therapy with sustained remission of urticaria and marked control of hyperphagia. While GLP1 receptor agonist therapy was offered, it was not a covered insurance benefit and the patient declined due to concern about additional injections, possible side effects, and medication costs.

## 7 Follow up and outcomes

### 7.1 Clinician and patient assessed outcomes

Both clinician and patient assessments demonstrated marked improvement after initiation of setmelanotide. The Hyperphagia Questionnaire for Clinical Trials (HQ-CT) score decreased from 20 to 0, reflecting resolution of hyperphagia and improved satiety control. Clinically, the patient achieved meaningful weight stabilization, and chronic idiopathic urticaria resolved within three weeks of therapy. Remission was sustained over a 10-month period, with only a single cold-induced episode. From the patient's perspective, quality of life improved significantly due to discontinuation of daily antihistamines, relief from recurrent hives, improved appetite control, and greater confidence in managing her health.

### 7.2 Important follow-up diagnostic and other test results

Follow-up testing emphasized the need for ongoing ophthalmologic evaluation to monitor reported night vision impairment, though this assessment remains pending due to insurance delays. Genetic counseling will also be crucial to understand the hereditary basis of BBS and considerations for this patient's children. Continued surveillance for renal involvement has been recommended given prior proteinuria, though renal function has remained stable. Other metabolic and autoimmune laboratory studies were unremarkable but ongoing monitoring will be highly recommended given the anticipated evolution of this syndrome.

### 7.3 Intervention adherence and tolerability

Adherence to therapy has been excellent, with the patient continuing setmelanotide daily and reporting consistent benefit. Tolerability was assessed through patient interviews and follow-up clinic visits, with the patient reporting improved control over appetite and hives, sustained energy, and satisfaction with treatment despite mild side effects.

### 7.4 Adverse and unanticipated events

The medication was generally well-tolerated. Transient nausea and fatigue occurred early in treatment but improved over time. A painful localized injection site reaction in January 2025 led to a brief pause in therapy, during which both hyperphagia and urticaria recurred. Symptoms resolved fully after restarting setmelanotide, and the reaction did not recur. The patient also developed moderate hyperpigmentation, which she was initially bothered by but has since deemed tolerable and incidentally facilitated dermatologic detection of skin cancers. No other adverse events have been observed.

## 8 Discussion

### 8.1 A scientific discussion of the strengths and limitations associated with this case report

The main strength of this case is the clear temporal relationship between setmelanotide administration and remission of chronic idiopathic urticaria (CIU), with symptom recurrence upon treatment interruption and resolution upon reinitiation, strongly supporting a therapeutic effect. Additionally, the patient had genetically supported Bardet-Biedl Syndrome (BBS), providing a well-defined disease context in which to assess the effects of melanocortin-4 receptor (MC4R) agonism. The patient's consistent follow-up and detailed clinical documentation strengthen the reliability of observed outcomes but lack of specialty consultation and insurance barriers made comprehensive multi-specialty care difficult. However, as a single case report, the ability to generalize these findings to broader populations is limited. Mechanistic insights were constrained by the absence of objective biomarkers of mast cell activation, such as serum tryptase or biopsy samples during urticaria flares. Pending ophthalmologic evaluation prevents complete phenotypic characterization, and the clinical contribution of variants of uncertain significance in KIDINS220 and *POMC* remains unclear. Furthermore, the possibility of coincidental remission or placebo response cannot be entirely excluded, and long-term durability of remission requires further study. Lastly, a limitation of this case is the high cost of setmelanotide therapy, which can exceed $300,000 annually in the United States [33] and may limit accessibility for many patients with Bardet-Biedl syndrome despite its demonstrated efficacy. This underscores the importance of ongoing efforts to improve insurance coverage and equitable access to rare-disease therapies.

### 8.2 Discussion of the relevant medical literature

Setmelanotide, an MC4R agonist, is FDA-approved for obesity due to rare genetic syndromes, including BBS, where it has shown significant improvement in appetite regulation and weight outcomes [[6], [7], [8]]. Although not described as major or minor features of BBS, studies have demonstrated that Bardet-Biedl syndrome (BBS) may also involve dermatologic manifestations and immune dysregulation [19,32]. Preclinical studies demonstrate that melanocortin signaling can regulate immune responses, with α-MSH analogs shown to suppress mast cell degranulation and allergic inflammation in murine models [12,13]. This aligns with the observed remission of CIU in our patient, a condition often resistant to antihistamines and sometimes requiring biologics such as omalizumab [12,13]. Moreover, recent evidence has linked CIU to autoimmune disease, underscoring immune dysregulation in its pathogenesis [21], [24]. Prior studies in BBS populations highlight the challenges of obesity management, often complicated by hyperleptinemia and leptin resistance [25], [26]. In this context, the patient's clinical response contributes to a growing body of literature on MC4R-targeted therapy for syndromic obesity and suggests potential immunomodulatory benefits. Moreover, genetic studies further emphasize the heterogeneity of obesity, with variants in *POMC* and *MC4R* contributing to severe early-onset forms [[27], [28], [29], [30], [31]].

### 8.3 Scientific rationale for conclusions

The observed remission of urticaria in temporal association with setmelanotide initiation, and recurrence upon drug interruption, provides a strong clinical rationale that MC4R agonism contributed to symptom control. BBS is characterized by ciliary dysfunction impairing GPCR trafficking and neuroendocrine signaling, leading to impaired satiety and metabolic dysregulation [4,5]. Setmelanotide corrects downstream signaling deficits by activating MC4R. Preclinical evidence shows that melanocortin peptides exert anti-inflammatory effects by modulating mast cell activity and cytokine pathways, providing a plausible mechanistic link between MC4R activation and improvement in CIU. While coincidental remission cannot be entirely ruled out, the reproducibility of symptom resolution upon reinitiation strengthens the hypothesis that setmelanotide directly influenced disease activity. d. Primary “take-away” lessons of this case report.

This case underscores the importance of recognizing rare genetic causes of obesity such as Bardet-Biedl Syndrome and highlights the potential for precision medicine to achieve benefits beyond weight regulation. The patient's resolution of refractory CIU with setmelanotide suggests that MC4R agonism may play a broader role in modulating mast cell–mediated inflammation. The key takeaway is that early identification of rare obesity syndromes and targeted therapy can yield meaningful improvements not only in metabolic control but also in comorbid immune-mediated conditions, reinforcing the need to document unexpected therapeutic benefits of novel agents to guide future research and clinical care.

## 9 Patient perspective

“Before this diagnosis, the impact on my life was huge. I battled my weight most of my life, struggling even when I did everything right. I dealt with depression, self-doubt, and unexplained medical issues that were often blamed solely on my weight, and I was told to just exercise more even though I was already pushing myself hard. My labs would come back abnormal for no clear reason, triggering more testing, and the hives were especially disruptive—I relied on daily Benadryl, which left me exhausted and unable to enjoy being outdoors with my family. Doctor after doctor dismissed me or passed me along, and it wasn't until Dr. Haggerty really dug deep that we finally reached the diagnosis. Looking back, I now recognize I had unexplained issues even as a child that were likely connected. When I finally received the diagnosis and began treatment, I felt like I could control my life again instead of my health problems controlling me. My confidence, mental health, and energy improved, I no longer battled cravings as much, my hives almost disappeared, and for the first time in years I was seeing real progress”.

“The side effects of Imcivree were noticeable at first, with nausea and one painful injection batch that made me pause treatment. During that break, the hives returned, confirming the medication was helping, and once I restarted, the benefits quickly returned. The hardest adjustment was the hyperpigmentation, which at first felt strange, but ultimately revealed skin cancers my dermatologist was able to treat—something that turned out to be a blessing. Through this journey, I've learned to advocate for myself, to never stop asking questions, and to see strength in documenting even the smallest details. While the thought of daily injections once seemed scary, the benefits have been life-changing. Thanks to Dr. Haggerty and her team, I feel healthier, happier, and more alive than I have in years, and I'm living life to its fullest”.

## 10 Informed consent

Written informed consent was obtained from the patient for publication of this case report and any accompanying images, including identifiable body parts.

## 11 Conclusion – key takeaway messages

● Early recognition of rare genetic causes of obesity, such as Bardet-Biedl Syndrome, is essential in guiding precision treatment.
● Setmelanotide provided effective control of both hyperphagia and refractory chronic idiopathic urticaria in this patient.
● MC4R agonism may play a broader role in modulating mast cell–mediated inflammation beyond weight regulation.
● Unexpected therapeutic benefits of novel agents should be documented to expand clinical knowledge and inform future research.

## Author contribution

Kate Haggerty: patient care, concept, writing, chart review, drafting, critical revision.

Ruth Abeles: writing, formatting, literature review, drafting, critical revision.

Jennifer Black: chart review, writing, formatting, literature review, critical revision.

All authors approved the final manuscript.

## Author disclosures

The authors declare no conflicts of interest related to this case report.

## Statement of AI use

Artificial intelligence (ChatGPT, 10.13039/100025178OpenAI) was used solely for grammar, language clarity, and formatting support during manuscript preparation. No content generation, data analysis, or interpretation was performed by AI. The authors take full responsibility for the scientific content and conclusions of this work.

## Source of funding

No specific funding was received for this work.

## Conflict of interest statement

Dr. Ruth Abeles and Dr. Jennifer Black report no conflicts of interest relevant to the content of this manuscript.

Dr. Kate Haggerty reports unpaid consulting involvement with Reltone and has professional affiliations with Advanced Diabetes Supply. These relationships are outside the scope of this work and did not influence the content or interpretation of this case report.
