# Supplementary material for: Resolution of chronic idiopathic urticaria with setmelanotide in a patient with Bardet-Biedl Syndrome: A case report
Source: Obes Pillars. 2025 Nov 3;16:100221. doi: 10.1016/j.obpill.2025.100221 (PMC12666839; doi:10.1016/j.obpill.2025.100221)
Supplement: Multimedia component 1 [file mmc1.docx]

| **Category** | **Criteria** | **Checklist for Patient** |
| --- | --- | --- |
| **Primary Features** | • Early-onset childhood obesity  • Hyperphagia  • Visual impairment  • Cognitive impairment, developmental delay  • Renal anomalies  • Genitourinary abnormalities  • Digit abnormalities (polydactyly, brachydactyly, etc) | ✅ Early-onset childhood obesity  ✅ Hyperphagia  ✅ Visual impairment (near-sightedness, low night vision)  ✅ Cognitive impairment in childhood  ✅ Renal anomalies (renal cysts, renal stones, recurrent proteinuria)  ✅ Genitourinary  abnormalities (PCOS) |
| **Secondary Features** | • Speech disorder/delay  • Dental anomalies  • Ataxia/poor coordination  • Cardiovascular anomalies  • Diabetes mellitus  • Hepatic fibrosis | ✅ Speech delay and stuttering as a child |

Table 1: The table below summarizes her phenotypic features aligned with BBS diagnostic guidelines

**Table 2.** Genetic testing results obtained from a saliva sample analyzed via next-generation sequencing through the Uncovering Rare Obesity program (Prevention Genetics, LLC). Three variants were identified: a heterozygous pathogenic variant in BBS9 (c.1120C>T; p.Arg374), previously reported in individuals with Bardet-Biedl Syndrome; a heterozygous variant of uncertain significance (VUS) in* KIDINS220 (c.4970G>A; p.Ser1657Asn), with limited evidence for a gene–disease relationship; and a heterozygous VUS in POMC (c.394C>G; p.Pro132Ala), which has been described in individuals with obesity but also observed in control populations, limiting interpretive significance.

| Gene | **DNA Variant** | **Protein Change** | **Zygosity** | **Classification** |
| --- | --- | --- | --- | --- |
| **BBS9** | c.1120C>T | p.Arg374* | Heterozygous | Pathogenic |
| **KIDINS220** | c.4970G>A | p.Ser1657Asn | Heterozygous | Variant of Uncertain Significance |
| **POMC** | c.394C>G | p.Pro132Ala | Heterozygous | Variant of Uncertain Significance |
