# Supplementary material for: Resolution of chronic idiopathic urticaria with setmelanotide in a patient with Bardet-Biedl Syndrome: A case report
Source: Obes Pillars. 2025 Nov 3;16:100221. doi: 10.1016/j.obpill.2025.100221 (PMC12666839; doi:10.1016/j.obpill.2025.100221)
Supplement: Multimedia component 2 [file mmc2.docx]

**Supplement 2: Images of Urticarial Lesions and Hand Edema**


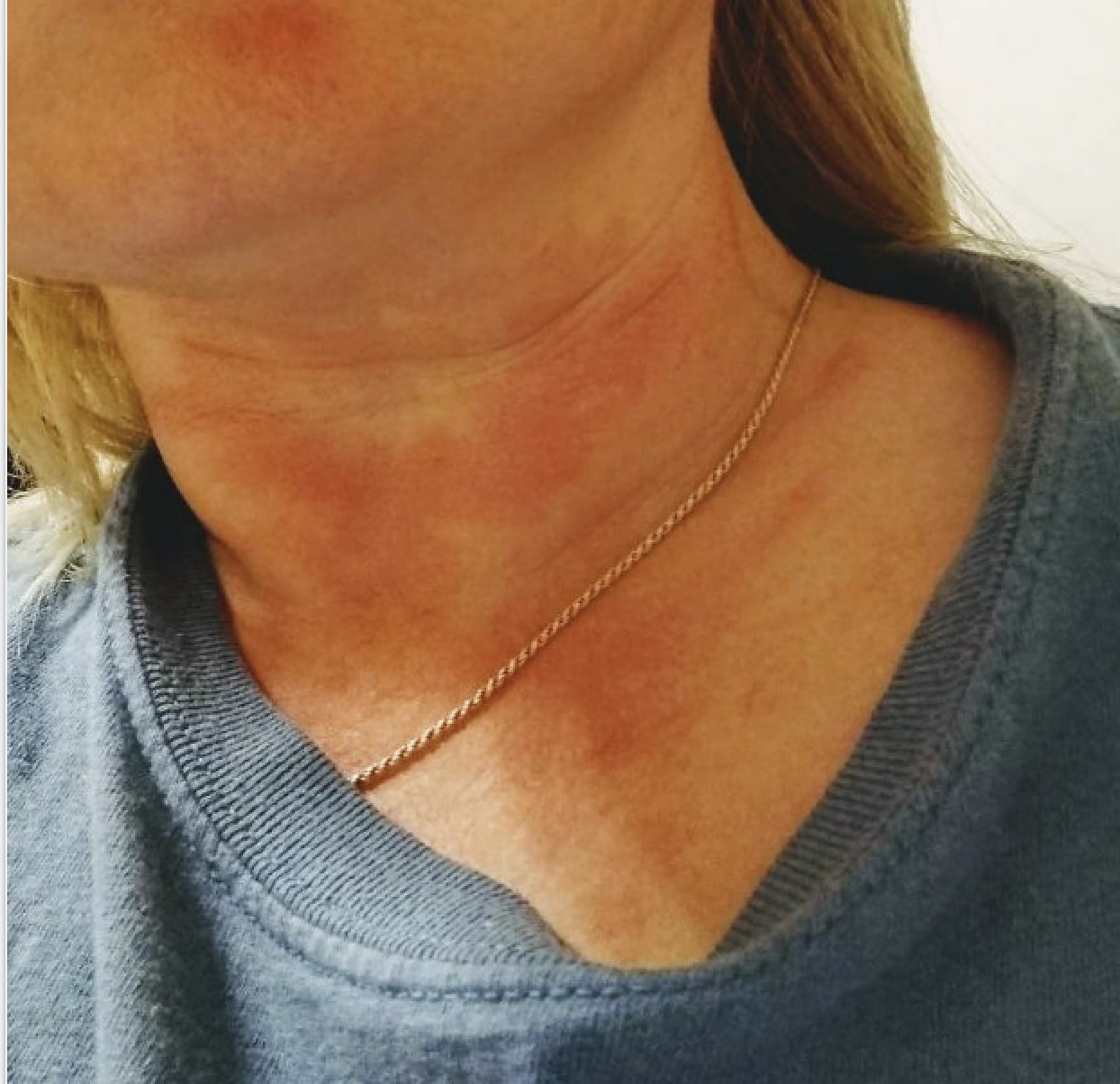


**Image 1.** Diffuse urticaria on the patient’s neck and torso (May 2022), demonstrating numerous erythematous, edematous wheals of varying diameter with irregular borders. The distribution and morphology are typical of acute urticarial eruptions.


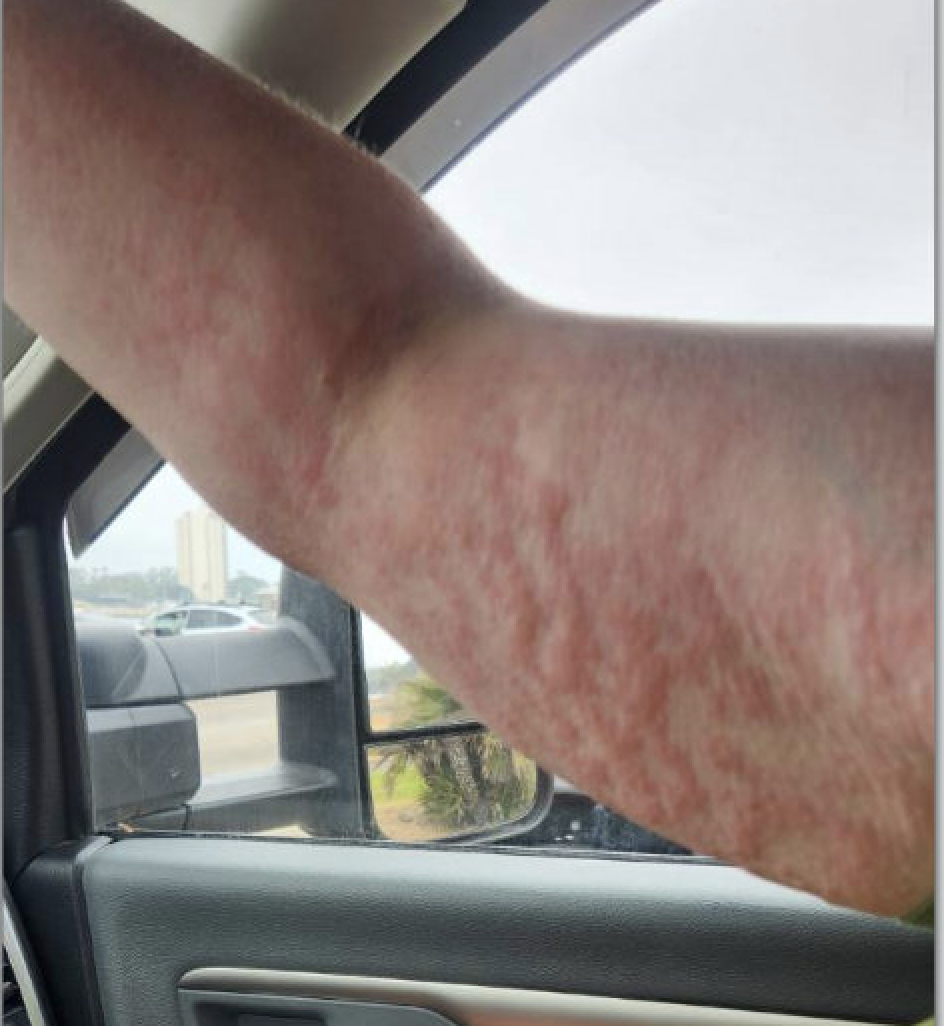


**Image 2.** Diffuse urticaria on the patient’s right arm (May 2022), demonstrating multiple erythematous, edematous wheals with irregular, undulating borders. The lesions vary in size and occasionally coalesce, consistent with the typical morphology of acute urticaria.


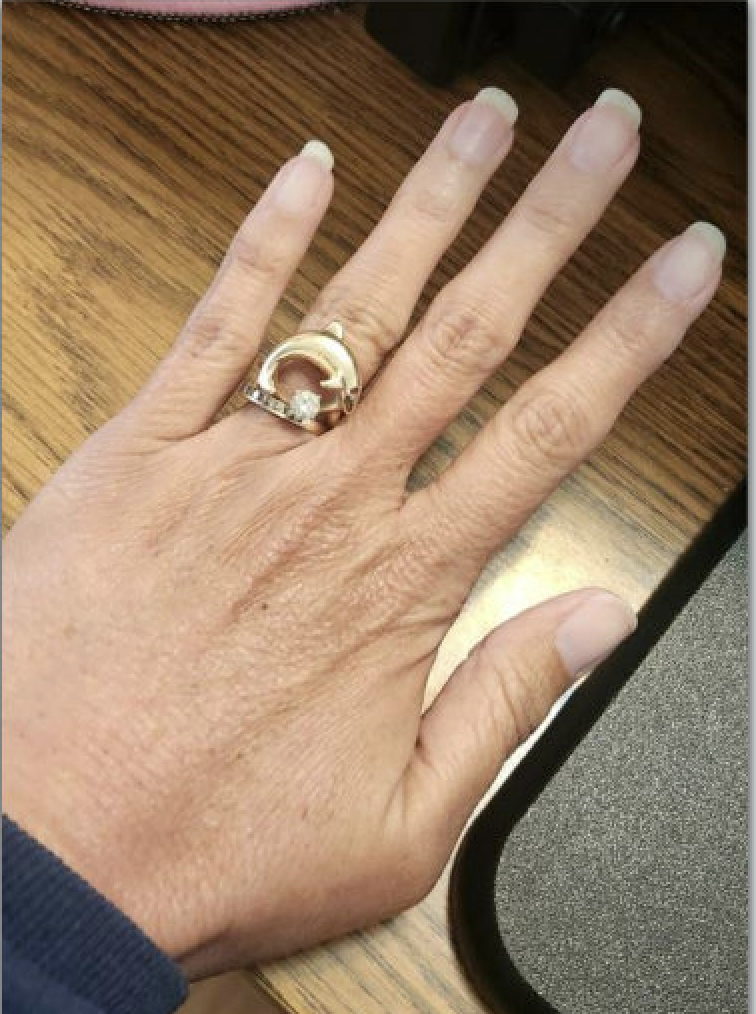

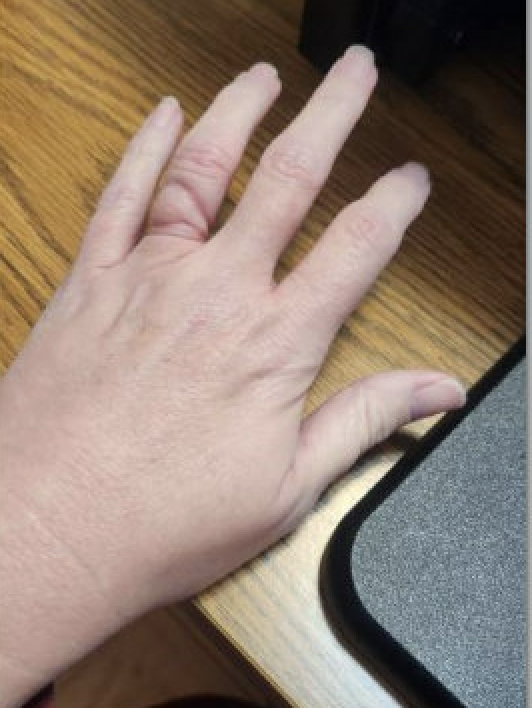


**Image 3.** A) *Baseline appearance of the left hand.* The photograph demonstrates the patient’s left hand with rings in situ. The skin surface appears intact without erythema, edema, or other visible abnormalities, representing the baseline condition prior to the allergic episode.

B) *Edematous changes with ring demarcation (May 2022)..* The same hand demonstrates diffuse soft tissue swelling involving the fingers, most pronounced at the sites of prior ring placement. Prominent indentation marks delineate the former ring margins, consistent with localized edema and pressure effect. The findings illustrate acute inflammatory changes and tissue edema occurring during the episode of diffuse urticaria.
